# Skeletal complications of brucellosis: A study of 464 cases in Babol, Iran

**Published:** 2017

**Authors:** Soheil Ebrahimpour, Masomeh Bayani, Zahra Moulana, Mohammad Reza Hasanjani Roushan

**Affiliations:** 1 Infectious Diseases and Tropical Medicine Research Center, Babol University of Medical Sciences, Babol, Iran.

**Keywords:** Brucellosis, Skeletal complication, Sacroiliitis, Spondylitis, Arthritis.

## Abstract

**Background:**

Skeletal complication of brucellosis is common in endemic region of brucellosis, but its frequency has not been clearly determined. The aim of this study was to determine the frequency of skeletal complication in brucellosis patients in Babol, north of Iran.

**Methods:**

From 2005-2015, 1252 cases of brucellosis were diagnosed at the Department of Infectious Diseases, Ayatollah Rouhani Teaching Hospital, in Babol, North of Iran. The diagnosis of brucellosis was established using serum agglutination test (SAT≥1/160), and 2-mercaptoethanol (2ME≥ 1/80) with clinical and epidemiological findings compatible with brucellosis. Among them, 464 (37.1%) cases demonstrated skeletal complication. The diagnosis of skeletal involvement was established with appropriate diagnostic facilities. The data were collected and analyzed.

**Results:**

The mean age of these patients (299 males, 165 females) was 33.2±17.6 years. Three hundred-thirty four (72%) cases were from rural areas. In 350 (75.4%) patients with peripheral arthritis, 242 (52.1%) cases were monoarthritis. Furthermore the knee arthritis was seen in 148 (31.9%) patients and hip in 54 (11.6) cases. Sacroiliitis was seen in 59 (12.7%) patients and spondylitis in 55 (11.9%) cases. There were no significant differences regarding the occurrence of these focal lesions in both sexes.

**Conclusion:**

The results show that about one-third of brucellosis in human is associated with skeletal complication. Peripheral arthritis, sacroiliitis and spondylitis are the frequent skeletal complications of human brucellosis.

**B**rucellosis is a global re-emerging zoonosis affecting animals and humans. It is apparent that this infection has posed great public health challenge for developing countries. Arabian Peninsula, Mediterranean basin, Indian subcontinent, Central and South America and South-Eastern Europe are the districts that have high prevalence of this infection (1, 2). The World Health Organization (WHO) estimates that half a million new cases of human brucellosis are reported annually (3). Brucellosis is endemic in all regions in Iran and its frequency was approximated from 0.5% to 10.9% in different areas in Iran (4). Complications of brucellosis can occur sometimes. It can involve any part of the body, such as the central nervous system (CNS), skeletal system, gastrointestinal (GI), genitourinary and cardiovascular system (5). Complications of human brucellosis of about 15% patients were recorded but osteoarticular engagements were reported between 10-25% of patients with arthritis and likely other organs can be involved (6-9).

The pattern of complications depends on the strain of Brucella, and complicated cases that are admitted to the hospitals. 

In this study, we describe skeletal complications and their frequencies in a large number of patients with brucellosis in North of Iran. 

## Methods

From 2005-2015, 1252 cases of brucellosis were diagnosed at the Department of Infectious Diseases, Ayatollah Rouhani Hospital, Babol University of Medical Sciences, North of Iran. Our treatment center covers more than 2 million people living in Mazandaran, North of Iran. The diagnostic criteria were clinical findings in accordance with standard agglutination test (SAT) ≥1/160 and 2-mercaptoethanol (2ME) ≥1/80 with clinical symptoms and signs compatible with brucellosis (10). We prepared a record for each patient's clinical symptoms and signs as well as laboratory finding were noted on it. 

The lab tests for these patients included complete blood count (CBC), erythrocyte sedimentation rate (ESR), rheumatoid factor (RF), C-reactive protein (CRP). Peripheral arthritis was the diagnosis on the clinical findings including swelling, effusion and limitation of motion in an involved joint and by using an x-ray and sonography sacroiliitis was diagnosed by clinical findings as well as bone scan or MRI. Spinal involvement was diagnosed using magnetic resonance imaging (MRI) in addition to clinical findings. We used appropriate diagnostic facilities in diagnosing other rare complications of brucella. 

The focal lesions between both sexes were compared. The data were collected and analyzed by SPSS version 16.Student’s t-test and chi-square tests were used when appropriate and a p-value <0.05 was considered significant.

## Results

During the study period, 464 (299 males and 165 females) cases with skeletal complications were reported and analyzed. The mean age of these patients was 33.2±17.6 years (range 15-80 years). Three hundred thirty-four (72%) patients were from rural areas. The clinical symptoms and signs of the patients are shown in table 1. The most common laboratory abnormalities in these patients were shown in table 2. 

**Table 1:** Demographic and clinical characteristics in patients with brucellosis according to complications

**Female** ** (n=165)**	**Male** **(n=299)**	**Number of Patients** **(n=464)**	**Signs and symptoms**
33.7±17.5	32.8±17.7	33.2±17.6	Mean±SD
			**Resident**
40 (30.8) 125(37.4)	90 (69.2) 209 (62.6)	130 (28.01) 334 (71.2)	Urban, no (%) Rural, no (%)
			**Symptoms**
70 (36.6) 67 (35.8) 65 (37.4) 54 (32) 57 (41.3) 77 (38.1) 5 (71.4)	121 (63.4) 120 (64.2) 109 (62.7) 115 (68.04) 81 (58.7) 125 (61.9) 2 (28.6)	191 (41.1) 187 (40.3) 174 (37.5) 169 (36.4) 138 (29.7) 202 (43.5) 7 (1.5)	Fever, no (%) Sweating, no (%) Chills, no (%) Malaise, no (%) Back pain, no (%) Arthralgia, no (%) Anorexia, no (%)
			**Signs**
8 (21.6) 5 (20)	29 (78.4) 20 (80)	37 (7.9) 25 (5.4)	Splenomegaly, no (%) Hepatomegaly, no (%)

**Table 2:** Laboratory and serological data in patients with focal complication

**patients**	**Parameter**
115 (24. 8)	Erythrocyte sedimentation rate >20 mm/h
147 (31.7)	CRP positive, no (%)
	WBC count, no (%)
16 (3. 4)	<4000 4000–9000 >9000
294 (63.4)	
154 (33.2)	
30 (6.5)	Alanine aminotransferase >40 IU/L, no (%)
13 (2.8)	RF positive, no (%)
5 (1.07)	Thrombocytopenia, no (%)
4 (0.9)	Thrombocytosis, no (%)

WBC: white blood cells, ALT: aspartate aminotransferase, CRP: C-reactive protein, ESR: erythrocyte sedimentation rate.

Peripheral arthritis was seen in 350 (75.4%) cases. Monoarthritis was seen in 242 (52.1%) patients mostly involved were knees 148 (31.9%) and hips 54 (11.6%). Monoarthritis was seen in 169 (36.4%) males and 73 (15.7%) females. Involvement of knee joint was seen in 105 (70.9%) males and in 43 (29.1%) females. There were no significant differences regarding the involvement of these lesions in both sexes. Sacroiliitis was seen in 59 (12.7%) patients and spondylitis in 55 (11.9%) cases. Other focal lesions are shown in table 3.

**Table 3:** Distribution of brucellar skeletal complications in 464 subjects and there differences between sexes

**Skeletal complication**	**Patients (N=464)** **No.%**	**Male** **No (%)**	**Female** **No (%)**
Peripheral arthritis	350 (75.4)	232(66.3)	118 (33.7)
Polyarthritis	50 (10.8)	32 (64)	18 (36)
Arthritis with artheralgia	58 (12.5)	31 (53.5)	27 (46.6)
**Monoarthritis (No=242)**			
Knee arthritis Hip arthritis Elbow Shoulder Sternoclavicular arthritis Wrist arthritis	148 (31.9) 54 (11.6) 5 (1.07) 3 (0.6) 21 (4.5) 11 (2.4)	105 (70.9) 38 (70.4) 4 (80) 3 (100) 12 (57.1) 7 (63.6)	43 (29.1) 16 (29.6) 1 (20) 0 (0) 9 (42.9) 4 (36.4)
Sacroiliitis	59 (12.7)	34 (57.6)	25 (42.4)
Spondylitis	55 (11.9)	33 (60)	22 (40)

* Complications between sexes were not significant.

## Discussion

Skeletal complication in brucellosisis is one of the serious medical problems and is frequentlyseen in endemic areas of infection. The common causative agent in our region is Brucella melitensis which causes the most severe form of disease with complications (11). The pattern and prevalence of complications depend on many factors for instance, strain of Brucella spp. and the length of the infection. The disease mainly affects various organs, particularly the musculoskeletal system as osteoarticular involvements (12). Osteoarticular involvement such as peripheral arthritis, sacroiliitis, osteomyelitis and spondylitis may disable and admit patients for several days. It should be noted that osteoarticular manifesetation of brucellosis happens frequently than arthritis (about 70%) (13). In this study, the most common sites of arthritis were the knees (31.9%) are followed by the hips (11.6%). Sternoclavicular arthritis (4.5%), wrist (2.4%), elbow (1.07%) and shoulder (0.6%), were the other forms of skeletal complications. Our findings were in agremeent with researchers (14, 15). The sacroiliac joints, hip and knee are the most commonly involved (14). In our study, monoarthritis of a peripheral joint was seen in 242 (52.1%) cases but a significant number of them had artheralgia 58 (12.5%) and myalgia together with monoarthritis. Arthritis in knee joint was the most frequently involved musculoskeletal site among localized complications of brucellosis. Although Brucella melitensis is a rare cause of Brucellar septic arthritis, it should be mentioned that either septic or non-septic arthritis are seen in both sexes in disease-endemic areas (16). As seen in this study, both genders were totally involved.

Overall in this study the knee, hip and sacroiliac joint were most frequently affected in adult males, whereas the knee and hip join were the most regularly affected areas in children (15). Arthritis in the upper extremiy joints like the elbow and wrist was unusual. These results were confirmed by other studies (17). 

In a previous study by Roushan et al., peripheral arthritis had been reported in 42 patients among 469 cases of brucellosis (9.2%) and the most frequent type was osteoarthricular involvement, and also Tas^,^ova et al. had shown that peripheral arthritis was the most frequent type of osteoarticular involvement (19.5%) which was in agreement with our results like other researchers in the endemic countries (18, 19). 

In other words, the rate of peripheral arthritis according to several studies was 14–19%. In our study, polyarthritis was seen in 50 (10.8%) patients with focal complications in both genders. Another study reported that polyarthritis was more common than any other types of skeletal involvement in females, but all types demonstrated a similar distribution in males (20). Sacroiliitis and spondylitis are the most common skeletal complications of brucellosis which have been seen in up to 80% of patients with focal complications (21). The rate of these complications was reported between 20-30% in the endemic areas (22). Moreover, according to the various findings, sacroiliitis constituted 48% of all the skeletal involvement in children compared to 60% in adult patients with brucellosis (23). 

These differences may be due to the strain of the organisms and the duration of the disease. Sacroilitis, spondylitis and large peripheral joint involvement may occur in brucellosis, particularly in young adults (24). In the present study, sacroiliitis and spondylitis were seen in 12.7%, and 11.9% cases, respectively. These percentages were comparable with the results of several reports regarding sacroiliitis (6%) and spondylitis (6.8%). These complications have reported more frequently in men than women (18). In some studies, 60% and 68% of brucellosis patients showed musculoskeletal complications that were higher than the finding of our research and sacroiliitis was the most common skeletal complications among them (10, 25). In regions where infection with Brucella melitensis predominates the sacroiliac joint is the most common site of involvement in the skeletal system (10). 

In conclusion, the findings demonstrate that about one-third of brucellosis in human is associated with skeletal complications. Peripheral arthritis, sacroiliitis and spondylitis are the most frequent skeletal complications of human brucellosis.

## References

[1] Hasanjani Roushan MR, Ebrahimpour S (2015). Human brucellosis: An overview. Caspian J Intern Med.

[2] Hasanjani Roushan MR, Bayani M, Soleimani Amiri S (2016). Evaluation of CD4+CD25+ FoxP3+ regulatory t-cells during treatment of patients with brucellosis. J Biol Regul Homeost Agents.

[3] de Figueiredo P, Ficht TA, Rice-Ficht A, Rossetti CA, Adams LG (2015). Pathogenesis and immunobiology of brucellosis: review of brucella–host interactions. Am J Pathol.

[4] Sofian M, Safaeipour L, Aghakhani A (2013). Screening of family members of patients with acute brucellosis in an endemic area of Iran. Iran J Microbiol.

[5] Solis Garcia del Pozo JSG, Solera J (2012). Systematic review and meta-analysis of randomized clinical trials in the treatment of human brucellosis. PloS One.

[6] Franco MP, Mulder M, Gilman RH, Smits HL (2007). Human brucellosis. The Lancet Infect Dis.

[7] Young EJ, Hasanjani Roushan MR, Shafae S, Genta RM, Taylor SL (2014). Liver histology of acute brucellosis caused by Brucella melitensis. Human Pathol.

[8] Kandasamy A, Ramalingam SK, Reddy BD, Krupananda H (2013). Anesthetic and hemodynamic management of a rare case of Brucella multivalvular endocarditis in cardiogenic shock undergoing emergency aortic valve replacement and mitral valve repair. Ann Card Anaesth.

[9] Guler S, Kokoglu OF, Ucmak H (2014). Human brucellosis in Turkey: different clinical presentations. J Infect Dev Ctries.

[10] Roushan MR, Amiri MJ, Laly A (2010). Follow-up standard agglutination and 2-mercaptoethanol tests in 175 clinically cured cases of human brucellosis. Int J Infect Dis.

[11] Hosseini SD, Azizpour M, Akbari N (2013). Amplification, cloning and expression of Brucella melitensis bp26 gene (OMP28) isolated from Markazi province (Iran) and purification of Bp26 Protein. Arch Razi Institut.

[12] Bozgeyik Z, Aglamis S, Bozdag PG, Denk A (2014). Magnetic resonance imaging findings of musculoskeletal brucellosis. Clin Imaging.

[13] Lulu AR, Araj GF, Khateeb MI (1988). Human brucellosis in Kuwait: a prospective study of 400 cases. Q J Med.

[14] Bosilkovski M, Zezoski M, Siskova D (2016). Clinical characteristics of human brucellosis in patients with various monoarticular involvements. Clin Rheumatol.

[15] Hasanjani Roushan MR, Amiri MJ (2013). Update on childhood brucellosis. Recent Pat Antiinfect Drug Discov.

[16] Solanki RR, Potikuri D, Rajasekhar L, Lakshmi V (2014). Septic Mono–arthritis of hip joint due to brucella melitensis: case report. Nat J Lab Med.

[17] Zamani A, Kooraki S, Mohazab Adabi R (2011). Epidemiological and clinical features of brucella arthritis in 24 children. Ann Saudi Med.

[18] Taşova Y, Saltoğlu N, Sahin G, Aksu HS (1999). Osteoarthricular involvement of brucellosis in Turkey. Clin Rheumatol.

[19] Hasanjani Roushan MR, Mohrez M, Smailnejad Gangi SM, Soleimani Amiri MJ, Hajiahmadi M (2004). Epidemiological features and clinical manifestations in 469 adult patients with brucellosis in Babol, Northern Iran. Epidemiol Infect.

[20] Geyik MF, Gur A, Nas K (2002). Musculoskeletal involvement of brucellosis in different age groups: a study of 195 cases. Swiss Med Wkly.

[21] Bozgeyik Z, Ozdemir H, Demirdag K (2008). Clinical and MRI findings of brucellar spondylodiscitis. Eur J Radiol.

[22] Hashemi SH, Keramat F, Ranjbar M (2007). Osteoarticular complications of brucellosis in Hamedan, an endemic area in the west of Iran. Int J Infect Dis.

[23] Sanaei Dashti A, Karimi A (2013). Skeletal involvement of brucella melitensis in children: a systematic review. Iranian J Med Sci.

[24] Logan LK, Jacobs NM, McAuley JB, Weinstein RA, Anderson EJ (2011). A multicenter retrospective study of childhood brucellosis in Chicago, Illinois from 1986 to 2008. Int J Infect Dis.

[25] Corderro-Sanchez M, Alvarez-Ruiz S, Lopez-Ochoa J, Garcia-Talavera JR (1990). Scintigraphic evaluation of lumbosacral pain in brucellosis. Arthritis Rheum.

